# Enhancing mu-ERD through combined robotic assistance and motor imagery: a novel approach for upper limb rehabilitation

**DOI:** 10.3389/fnhum.2025.1571386

**Published:** 2025-06-04

**Authors:** Hiroki Yasuda, Masaya Ueda, Keita Ueno, Yasuo Naito, Ryouhei Ishii, Takashi Takebayashi

**Affiliations:** ^1^Department of Occupational Therapy, Osaka Metropolitan University Graduate School of Rehabilitation Science, Habikino, Japan; ^2^Therapy Department, Takarazuka Rehabilitation Hospital, Takarazuka, Japan

**Keywords:** motor imagery, robotic therapy, mu-ERD, stroke rehabilitation, EEG, sensorimotor cortex

## Abstract

**Introduction:**

Previous research has suggested that mu-event-related desynchronization (mu-ERD) reflects neural activity associated with motor observation and execution, primarily within the sensorimotor cortex. This study aimed to investigate the effects of combining robotic full-assist therapy with motor imagery on mu-ERD in healthy adults for potential application in stroke patients with severe upper limb paralysis.

**Methods:**

Fifteen healthy adults were included in this study. Each participant performed three conditions using the ReoGo-J® robotic system: voluntary movement, full-assist robotic therapy without motor imagery, and full-assist robotic therapy with motor imagery. Electroencephalography (EEG) was used to measure mu-ERD, focusing on the 8–10 Hz and 10–13 Hz frequency bands at the C3, C4, Cz, and Pz electrodes.

**Results:**

Significant differences in mu-ERD occurrence were observed at C3 (8–10 Hz) and C4 (10–13 Hz) between the conditions. The combination of motor imagery and robotic therapy demonstrated a higher frequency of mu-ERD occurrence than the other conditions, with moderate effect sizes. However, no significant differences in mu-ERD attenuation rates were found between the conditions. This suggests variability in individual responses.

**Discussion:**

These findings highlight the potential of robotic full-assist therapy combined with motor imagery to stimulate neural mechanisms associated with motor recovery. Future studies should include a larger sample size and patients with stroke to validate these findings and explore their clinical applications.

## 1 Introduction

A Cochrane review of 16 randomized trials showed that motor observation after stroke enhances upper limb function by promoting neuroplasticity via the mirror neuron system (MNS) (Borges et al., 2022). Mu-event-related desynchronization (mu-ERD) is characterized by a decrease in the power of the mu rhythm (8–13 Hz) over the sensorimotor cortex during voluntary movement, motor imagery, or action observation. This desynchronization reflects the activation of sensorimotor neurons and is believed to be mediated by the MNS, which links perception and action through common neural substrates (Heida et al., 2014; Fox et al., 2016). From a clinical perspective, mu-ERD has emerged as a promising biomarker in neurorehabilitation, especially for evaluating motor-related cortical activity in individuals with neurological disorders, such as stroke or Parkinson’s disease (Abiri et al., 2019; Remsik et al., 2019). In particular, its application in brain–computer interface (BCI) systems enables real-time detection of motor intention and adaptive rehabilitation strategies based on cortical responses. The mu rhythm, observed during action observation or execution, induces event-related desynchronization (ERD), i.e., mu-ERD. Mu-ERD reflects MNS activity (Fox et al., 2016), which is a neural network activated during action observation and execution (Rizzolatti and Craighero, 2004). This interpretation is further supported by studies showing that individuals with impaired MNS function, such as patients with Parkinson’s disease, demonstrate reduced mu-ERD responses during movement observation compared with healthy controls (Heida et al., 2014). These findings reinforce the idea that mu-ERD can serve as a neurophysiological marker of MNS activity. Interventions that produce mu-ERD include mirror therapy (Lee et al., 2015), imagery therapy (Kruse et al., 2020), robot therapy (Formaggio et al., 2013), robot therapy combined with mirror and imagery therapies (Formaggio et al., 2013; Park et al., 2020), and functional electrical stimulation (Remsik et al., 2019).

In a previous study investigating mu-ERD, Tangwiriyasakul et al. (2014) reported that stroke patients with larger and more extensive ERD on the injured side of the brain showed improvement in Fugl–Meyer Assessment (FMA) scores after performing motor execution and motor imagery of hand opening and closing movements. A treatment method using mu-ERD is electroencephalography (EEG)-based BCI. This approach promotes neuroplasticity by detecting mu-ERDs in EEG and operating a rehabilitation robot accordingly (Abiri et al., 2019). Furthermore, Remsik et al. (2019) reported that an intervention combining EEG-based BCI with functional electrical stimulation in patients with stroke resulted in increased mu-ERD and a concomitant improvement in functional connectivity between the ipsilateral primary motor cortex and the ipsilateral premotor cortex. This suggests that mu-ERD may contribute to improved motor function and altered neuroplasticity in patients with stroke.

In Japan, the ReoGo-J® robotic system (Teijin pharma, Osaka, Japan) is widely used for upper limb rehabilitation after stroke. Its effectiveness has been validated in several randomized controlled trials involving stroke patients with moderate to severe upper limb paralysis (Takahashi et al., 2016; Takebayashi et al., 2022). However, its effectiveness for stroke patients with severe upper limb paralysis has not been sufficiently investigated. Meta-analyses of other robotic systems worldwide have reported no significant improvement in FMA scores in stroke patients with severe upper limb paralysis (FMA ≤ 21) compared with controls, suggesting that conventional passive robotic therapy may not be effective in such cases (Zhang et al., 2022).

Regarding the effects of combining motor imagery and robotic therapy, several individual studies included in the meta-analysis by Kruse et al. (2020) incorporated BCI with motor imagery–combined robotic therapy to support motor intention. These studies reported improvements in upper extremity motor function and changes in resting-state functional connectivity, suggesting possible correlations with motor recovery.

On the basis of these reports, we hypothesized that combining robotic rehabilitation with motor imagery would be more effective than conventional robotic therapy alone. Therefore, we aimed to develop a novel therapy for stroke patients with severe upper limb paralysis, combining passive upper limb movements via robotic assistance with motor imagery, and to examine its effects on mu-ERD. In the present study, we compared the frequencies of mu-ERDs in healthy adults under different conditions to test whether the full-assist condition combined with motor imagery may have a different effect on mu-ERD compared with the full-assist condition without voluntary movement or imagery. If successful, this method may provide a more effective means of restoring upper limb function in patients with severe stroke.

## 2 Materials and methods

### 2.1 Ethical considerations

This study was approved by the Ethical Review Board of Osaka Prefectural University (approval number: 2022–203). All participants provided verbal and written informed consent prior to participation. The study involved non-therapeutic, robot-assisted passive movements and EEG recordings in healthy adults. As this was a non-interventional study that did not involve any therapeutic or diagnostic procedures, clinical trial registration was not required under current Japanese regulations.

### 2.2 Participants

The study participants were healthy employees of the Takarazuka Rehabilitation Hospital who were at least 20 years old at the time of consent, were determined to be right-handed by the Edinburgh Handedness Inventory, and had no visual or mental impairment. Previous studies included in the meta-analysis of mu-ERD by Fox et al. (2016) often had a sample size of approximately 10; the sample size in the present study was set to 15, with reference to previous studies (Fox et al., 2016).

### 2.3 Experimental procedure

#### 2.3.1 ReoGo-J^®^ robotic system

ReoGo-J^®^ (Teijin Pharma) is an end-effector type robotic system for shoulder and elbow rehabilitation, featuring 17 different exercise tasks, 5 levels of movement assistance modes, and 3 levels of adjustable exercise load. During this robotic therapy, the tasks are displayed on a monitor in front of the patient who is then instructed to perform them.

#### 2.3.2 Paper evaluation

The Edinburgh Handedness Inventory was used to determine right-handedness, a prerequisite for this study (Oldfield, 1971).

The Japanese version of the Kinesthetic and Visual Imagery Questionnaire (KVIQ) was used to assess visuomotor imagery and kinesthetic abilities (Nakano et al., 2018). The KVIQ is a standardized tool that rates imagery clarity and intensity on a five-point scale. It offers a simple and practical assessment of motor imagery ability and is available in two formats: the 20-item (KVIQ-20) and the 10-item versions. The Japanese version of the KVIQ, translated from the original, has been validated for reliability and utility in motor learning and rehabilitation. To assess motor imagery performance, participants completed the upper extremity items (questions 1–5) of the KVIQ-20 after each imagery task. The maximum total score for each task was 25 points. After completing all tasks, participants were asked additional questions. First, they rated how clearly they were able to visualize the motor imagery task on an 11-point scale (0–10), with 10 indicating a vivid voluntary movement. Then, they were asked whether they used kinesthetic imagery, visual imagery, or both. Lastly, participants who performed visual imagery were asked whether the imagery was from a first-person or third-person perspective.

#### 2.3.3 EEG measurement

EEG data were recorded using the EEG-9100 Neurofax μ encephalograph (Nihon Kohden, Tokyo, Japan). Nineteen channels (Fp1, Fp2, F7, F3, Fz, F4, F8, T7, C3, Cz, C4, T8, P7, P3, Pz, P4, P8, O1, and O2) were used for measurement, according to the international 10–20 method. An earlobe served as the reference electrode, and measurements were performed with participants in a sitting position. Although the room in which the EEG data were measured was not shielded, measurements were taken after confirming the absence of noise, and we maintained a sufficient distance from noise-sensitive equipment.

#### 2.3.4 Experimental tasks

**Figure 1:**
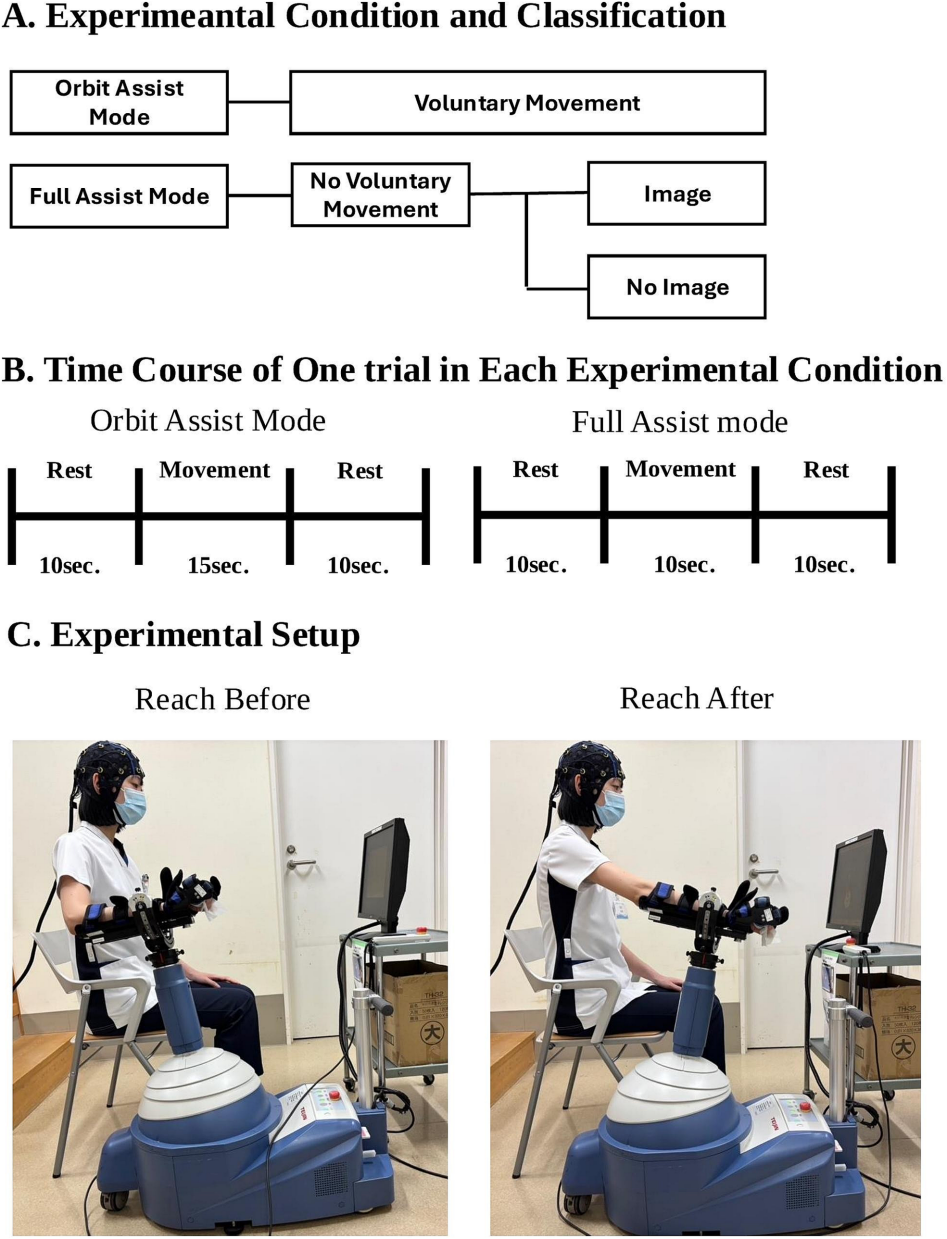
Overview of the Experimental Conditions, Trial Timing, and Setup. **(A)** Overview of the experimental conditions. The study consisted of three conditions: voluntary movement (Orbit Assist Mode), full-assist robotic movement without voluntary control, and full-assist robotic movement with motor imagery. **(B)** Time course of each trial. Orbit Assist Mode trials included 15 s of movement and 10 s of rest; Full Assist Mode trials included 10 s of movement and 10 s of rest. **(C)** Photographs showing a participant using the ReoGo-J® robotic system before and after the reaching task.

The robotic system was set to Orbit Assist Mode for voluntary movements and full-assist mode without voluntary movements. For both conditions, the task was forward-reaching, and the number of sessions was 30. Each orbit-assist session (with voluntary movement) included 10 s of rest, 15 s of practice, and 10 s of rest, whereas full-assist sessions (without voluntary movement) included 10 s of rest, 10 s of exercise, and 10 s of rest. The longer practice time in the orbit-assist condition accommodated the variable timing of exercise completion due to voluntary movement. The full-assist mode was performed with and without imagery (Figure 1). In the imagery condition, participants were instructed to perform motor imagery of the forward-reaching task without any actual muscle contraction. In contrast, the non-imagery condition involved neither voluntary movement nor mental imagery. Am electromyograph (Trunk Solution, Tokyo, Japan) was used to detect voluntary movements during the full-assist task. To minimize order effects, the order of presentation of the three conditions (Orbit Assist Mode, full-assist mode with imagery, and full-assist mode without imagery) was randomized for each participant, which prevented consistent presentation of a particular condition at the beginning or end of the testing sequence.

#### 2.3.5 Data analysis

EEG data were recorded at a sampling rate of 500 Hz, and the collected data were bandpass filtered using BESA Research software (BESA, Gräfelfing, Germany) with a low-cut of 2 Hz and a high-cut of 50 Hz. Independent component analysis was performed using the EEGLAB toolbox (MathWorks, Natick, MA, USA) for noise reduction and signal processing, employing the “Runica” algorithm. Each Independent Component was visually checked, and components corresponding to blinking, eye movement, and occipital myoelectric activity were removed before reconstructing the EEG data. Fast Fourier transform was used for the frequency analysis and performed every 2.05 s.

Pineda (2005) identified distinct characteristics between low-frequency (8–10 Hz) and high-frequency (10–13 Hz) mu-ERDs. It was reported that low-frequency mu-ERD responds nonspecifically to movement in general, regardless of specific movement types, whereas high-frequency mu-ERD exhibits more specific patterns, such as in response to finger movements. Because the robotic system used in this study targets the shoulder and elbow joints, and considering the differences in reactivity between high and low frequencies, mu-ERD was divided into low-frequency and high-frequency bands for analysis. The sites chosen for analysis were Cz, C3, C4, and Pz, following the international 10–20 method. This selection was hypothesis-driven, focusing on electrodes overlying the sensorimotor cortex, the primary region known to generate mu rhythm and exhibit mu-ERD during upper limb motor execution and imagery (Pineda, 2005; Pfurtscheller et al., 2006; Formaggio et al., 2013; Li et al., 2022). This approach aligns with established literature investigating mu-ERD phenomena in similar contexts. Our primary aim was to quantify mu-ERD modulation specifically at these key sensorimotor sites across the experimental conditions. According to previous studies, the mu-ERD decay period was considered to begin 2 s before the start of exercise, and the pre-attenuation period was set during 5–8 s of rest. Mu-ERD occurs during exercise (Fox et al., 2016). Thus, to account for the influence of the robotic system’s automatic and fully assisted movements during exercise on mu-ERD, the post-decay period was set to the same duration as the resting state, specifically 0–3 s after exercise initiation. Although the duration of movement varied between conditions (e.g., 15 s for voluntary movement versus 10 s for full-assist conditions), this difference was necessary to accommodate the variable completion time of voluntary reaching movements. Importantly, this variation did not influence the EEG analysis, as the mu-ERD evaluation window was uniformly set to the initial 0–3 s after movement onset across all conditions. This consistent time window allowed for appropriate comparisons of neural responses, minimizing any potential confounding effects of movement duration. Additionally, the presence or absence of mu-ERD was treated as a categorical variable and compared among the three experimental conditions using the chi-squared test, which is appropriate for analyzing differences in proportions across multiple groups. The effect size was calculated using Cramér’s V.

In contrast, the attenuation rate of mu-ERD—a continuous variable—was analyzed using a one-way analysis of variance (ANOVA) to compare means across the three conditions: voluntary movement, full-assist with imagery, and full-assist without imagery. Tukey’s honestly significant difference was used for *post hoc* comparisons. This analytical approach is standard when handling continuous outcome measures in multi-group comparisons.

Because previous studies examining the frequency and magnitude of mu-ERD across these three specific conditions are limited, we selected conventional and statistically appropriate methods based on variable type. The significance level for all statistical tests was set at *p* < 0.05, and η^2^ was used to estimate the effect size for ANOVA.

Because the study sample size was determined on the basis of prior research, a post hoc power analysis was conducted to verify its appropriateness using the obtained results. The power values before and after each decay were calculated, and Shapiro–Wilk’s test was used to assess the normality of the data. The paired t-test or Mann–Whitney’s U test was used for normally and non-normally distributed data, respectively, to determine whether post-decay values were significantly lower than pre-decay values; a significant difference indicated the occurrence of mu-ERD.

## 3 Results

Fifteen healthy adults (11 males and 4 females) were recruited, with a mean age of 25.8 ± 3.1 years and a mean Edinburgh Handedness Inventory score of 91.2 ± 10.8, confirming right-handedness in all participants. On the KVIQ, visual imagery scored 18.9 ± 2.9 and kinesthetic imagery scored 16.7 ± 3.8 (Table 1). After all the tasks were completed, the average clarity of the motor imagery task was 4.66 ± 1.17. Regarding the type of motor imagery, 14 participants used kinesthetic and visual imagery, and 1 participant used only visual imagery. Of those who performed visual imagery (n = 15), 13 participants performed imagery from a first-person perspective and 2 from a third-person perspective.

**Table 1:** Participants’ characteristics.

Sex (n)	11 males; 4 females
Age (years)	25.8 ± 3.1
Edinburgh Handedness Inventory score (points)	91.2 ± 10.8
Kinesthetic and Visual Imagery Questionnaire score (points)	Visual Imagery: 18.9 ± 2.9 Kinesthetic imagery: 16.7 ± 3.8

EMG recordings detected no muscle contraction during all tasks with and without motor imagery under assisted conditions. The chi-squared test showed significant differences at C3 and C4 at 8–10 Hz and 10–13 Hz, respectively, but not at the other electrodes. Using Cramér’s V, the effect sizes for 8–10 Hz were as follows: C3, moderate (V = 0.436); C4, moderate (V = 0.166); Cz, moderate (V = 0.276); and Pz, not relevant (V = 0.063). The effect sizes for 10–13 Hz were as follows: C3, small (V = 0.109); C4, moderate (V = 0.387); Cz, small (V = 0.131); and Pz, small (V = 0.259) (Table 2). One-way ANOVA for C3 (8–10 Hz) and C4 (10–13 Hz), which were found to be significantly different according to the chi-squared test result, revealed no significant differences; the effect sizes were η^2^ = 0.106 for C3 (8–10 Hz) and η^2^ = 0.085 for C4 (10–13 Hz), both classified as medium. Tukey’s multiple comparison test showed no significant differences for any combination. Power analysis showed that the chi-squared test results had powers of 74.8% at C3 (8–10 Hz) and 63.8% at C4 (10–13 Hz), which was sufficiently adequate for C3. In contrast, one-way ANOVA results had powers of 27.05% for C3 (8–10 Hz) and 23.7% for C4 (10–13 Hz), indicating insufficient power for both (Table 3).

**Table 2:** Occurrence and effect size of mu-ERD (8–13 Hz) by frequency band, electrode site, and task condition.

Electrode	Freq. Band	Task type	Occurrence (*n*)	Non-occurrence (*n*)	*p*-value	Effect size (Cramér’s V)
C3	8–10 Hz	Voluntary	4	11	**0.014**	**0.436**
		Image	12	3		
		No Image	8	7		
	10–13 Hz	Voluntary	7	8	0.77	0.109
		Image	9	6		
		No Image	8	7		
C4	8–10 Hz	Voluntary	6	9	0.54	0.166
		Image	9	6		
		No Image	7	8		
	10–13 Hz	Voluntary	6	9	**0.034**	**0.387**
		Image	12	3		
		No Image	5	10		
Cz	8–10 Hz	Voluntary	6	9	0.18	0.276
		Image	11	4		
		No Image	8	7		
	10–13 Hz	Voluntary	9	6	0.68	0.131
		Image	11	4		
		No Image	9	6		
Pz	8–10 Hz	Voluntary	8	7	0.91	0.063
		Image	9	6		
		No Image	8	7		
	10–13 Hz	Voluntary	12	3	0.22	0.259
		Image	8	7		
		No Image	8	7		

Freq. Band, Frequency Band. “Voluntary” refers to Orbit Assist Mode with voluntary movement. “Image” refers to full-assist robotic therapy with motor imagery. “No image” refers to full-assist robotic therapy without motor imagery. p-values and effect sizes (Cramér’s V) are based on chi-squared tests. Statistically significant results (*p* < 0.05) are shown in bold.

**Table 3:** Mu-ERD attenuation rates and post hoc comparisons.

Electrode	Freq.Band	Task type	Attenuation Rate (mean ± SD)	*n*	ANOVA *p*-value	Effect size (η^2^)	Tukey’s post hoc test		
							V vs. I	V vs. NI	I vs. NI
C3	8–10 Hz	Voluntary	−0.25 ± 0.12	4	0.31	0.106	0.60	0.29	0.68
		Image	−0.32 ± 0.15	12					
		No image	−0.37 ± 0.07	8					
C4	10–13 Hz	Voluntary	−0.25 ± 0.14	9	0.36	0.085	0.46	0.42	0.94
		Image	−0.33 ± 0.17	12					
		No image	−0.36 ± 0.09	15					

Freq. Band, Frequency Band. Tukey’s post hoc test p-values are presented for all pairwise comparisons: V, Voluntary movement, I, Imagery with robotic assistance, NI, No imagery with robotic assistance. Statistically significant values (*p* < 0.05) are shown in bold. η^2^ = partial eta squared (effect size).

## 4 Discussion

In this study, we investigated the effects of combining fully assisted upper extremity exercise and imagery therapy with robotic therapy using a robotic system on mu-ERD in healthy adults. Three conditions were assessed: robotic therapy with voluntary movement, robotic therapy with full-assisted movement and motor imagery, and robotic therapy with full-assisted movement only. The results showed that combining fully assisted robotic therapy with motor imagery produced the highest number of mu-ERDs at C3 (8–10 Hz) and C4 (10–13 Hz) compared with the other two conditions. Significant differences in frequency were observed between the groups, with moderate effect sizes as indicated by Cramér’s V. No statistically significant differences were found in the attenuation rates of mu-ERD before and after exercise across the three conditions, and the magnitude of mu-ERD was comparable.

Mu-ERD is generated in the sensorimotor cortex (Pineda, 2005). Li et al. (2022) previously used mu-ERD detected at C3 and C4 in a BCI study combining imagery tasks with full-assist robotic therapy and other modalities in patients with stroke, which are the same sites identified in the present study.

Pfurtscheller et al. (2006) reported that a mu-ERD of 8–10 Hz was observed in contralateral brain regions during hand movement imagery. Furthermore, Frenkel-Toledo et al. (2014) investigated MNS activation through low-frequency mu-ERD during hand movement observation in patients with stroke. They reported contralateral activation during right-hand observation, suggesting that low-frequency mu-ERD may serve as a physiological marker reflecting MNS activity. Based on these findings, the responses at C3 (8–10 Hz) observed in the present study may reflect MNS activity underlying motor imagery.

Hasegawa et al. (2017) reported bilateral mu-ERD during shoulder motor imagery in healthy adults. They also observed increased motor evoked potentials in shoulder muscles associated with ipsilateral mu-ERD, which may reflect the excitability of non-crossing pathways. It is possible that the responses observed at C4 (10–13 Hz) in the current study were elicited by excitation of these non-crossing pathways in the shoulder induced by imagery of shoulder joint movement.

Individual variability in mu-ERD responses may also be shaped by neurochemical and inflammatory factors. For example, brain-derived neurotrophic factor, which plays a critical role in neural plasticity and excitability, fluctuates in response to neural activation and pathological conditions, such as seizures (Poniatowski et al., 2021). Moreover, inflammation-related proteins, such as progranulin, have emerged as modulators of neuronal function through their regulatory roles in immune signaling pathways (Saeedi-Boroujeni et al., 2023). Although our study involved only healthy participants, such molecular influences may partly explain inter-individual differences in mu-ERD modulation, even under non-pathological conditions.

This study found no statistically significant difference in mu-ERD attenuation rates between C3 (8–10 Hz) and C4 (10–13 Hz) under any condition, although a moderate effect size was observed. While not statistically significant, this may indicate a potentially meaningful difference between conditions. However, large variations in individual decay rates and imbalances in the number of participants exhibiting mu-ERD in each condition may have influenced the results. Variation in responses and unequal sample sizes likely reduced statistical power. This may explain the lack of significant group differences. In other words, although no statistically significant differences were observed in the magnitude of the attenuation rate, the effect size results suggest that a meaningful difference cannot be ruled out. Future studies should adopt larger sample sizes to address inter-individual variability and improve statistical power.

This study has several strengths. It is the first to examine the effects of ReoGo-J® full-assist training combined with imagery therapy on mu-ERD occurrence in healthy adults. It also confirmed MNS activation in the contralateral cortex via low-frequency mu-ERD and ipsilateral activation from shoulder imagery via high-frequency mu-ERD.

However, this study also has limitations. First, although the order of conditions was randomized to minimize order effects, such effects could not be entirely excluded. Second, the sample size (n = 15) was based on those in prior studies, many of which used approximately 10 participants. *Post hoc* power analysis using the chi-squared test showed a power of 74.8% for the C3 electrode, indicating a certain level of reliability. However, the chi-squared test and ANOVA for the C4 electrode yielded power below 80%, suggesting insufficient statistical power under some conditions. Future studies should adopt larger sample sizes to enhance reliability. Third, since this study included only healthy adults, it is uncertain whether similar effects would occur in patients with stroke. Furthermore, the absence of mu-ERD in some participants suggests individual differences in response to the combined therapy. Future research should investigate this intervention in populations with stroke to determine appropriate clinical applications. Fourth, while EEG data were recorded from 19 channels, our primary analysis focused *a priori* on specific electrodes overlying the sensorimotor cortex (C3, C4, Cz, Pz) to directly address our hypotheses regarding mu-ERD modulation at its principal source. Consequently, we did not perform topographic analyses to visualize the broader spatial distribution of neural activity across the scalp. While our findings quantify mu-ERD changes at key sensorimotor sites, such topographic mapping requires analysis across a wider electrode array and could provide valuable insights into the spatial dynamics and potential involvement of distributed networks, such as the MNS. Future analyses leveraging the full recorded dataset could explore these spatial aspects.

Taken together, these results suggest that combining full-assist robotic therapy with motor imagery therapy for the shoulder and elbow joints facilitates mu-ERD generation and elicits neural responses consistent with those previously reported for motor imagery therapy alone. Therefore, robotic full-assist therapy combined with motor imagery may represent an effective rehabilitation approach for the shoulder and elbow joints.

## Data Availability

The raw data supporting the conclusions of this article will be made available by the authors, without undue reservation.
